# How Far Have We Come in the Field of Nerve Regeneration After Trigeminal Nerve Injury?

**DOI:** 10.1007/s40496-016-0115-x

**Published:** 2016-09-13

**Authors:** Annika Rosén, Arezo Tardast, Tie-Jun Shi

**Affiliations:** 1 Division of Oral and Maxillofacial Surgery, University of Bergen, Årstadveien 19, 5020 Bergen, Norway; 2 Department of Clinical Dentistry, University of Bergen, Årstadveien 19, 5020 Bergen, Norway; 3 Department of Oral and Maxillofacial Surgery, Södra Älvsborg Hospital, 501 82 Borås, Sweden; 4 Department of Biomedicine, University of Bergen, Bergen, Norway

**Keywords:** Sensory neurons, Orofacial sensory disturbances, Trigeminal nerve, Inferior alveolar nerve, Lingual nerve, Infraorbital nerve, Hyperalgesia, Hypoasthesia, Nerve regeneration, Mesenchymal stem cells, Adipose derived stem cells, Stem cells therapy

## Abstract

Patients suffering from nerve injury with sensory disturbances or orofacial pain have greatly reduced quality of life, and it is a big cost for the society. Abnormal sensations caused by trigeminal nerve injury often become chronic, severely debilitating, and extremely difficult to treat. In general, non-invasive treatment such as drug treatment has been insufficient, and there are currently few available effective treatments. Surgical interventions such as end-to-end connection or nerve grafting have disadvantages such as donor site morbidity or formation of neuroma. There is need for optimizing the technique for nerve repair, especially for the trigeminal nerve system, which has so far not yet been well explored. Recently, tissue engineering using biodegradable synthetic material and cell-based therapies represents a promising approach to nerve repair and it has been reported that mesenchymal stem cell (MSC) has an anti-inflammatory effect and seems to play an important role in nerve healing and regeneration.

## Introduction

### Background

Dysfunction of the trigeminal nerve due to trauma, diseases, or unknown causes is for the patients distressing. The sensory disturbances and/or pain are unpleasant conditions. It can involve the function of the mandible, the muscles, the skin of chin and lips, the intra orally mucosa, and the tongue and give rise to several problems such as pain, inability to move the jaw, tongue-lip or cheek biting, inability to maintain food and liquid competence, burning sensation with provocative stimuli, change in speech pattern, and change in taste perception [1]. Studies of patients that are affected by temporomandibular disorders (TMD), which may present a longstanding pain condition where muscles and/or joint are involved, have revealed that psychological factors dominate as a consequence of living with pain [2]. In a study where TMD patients were investigated with a multidisciplinary approach, most of the patients had a long history of pain, significant high levels of catastrophizing, and high occurrence of anxiety and/or depression [3]. Injury to the somatosensory pathways may either increase the nerve transmission like in allodynia and hyperalgesia or decrease the transmission such as in hypoesthesia or anesthesia [4, 5]. An important sequel of nerve injury and other nervous system diseases is neural degeneration. Abnormal sensation induced by peripheral nerve injury has been considered as a progressive neurodegenerative disease [6].

**Fig. 1:**
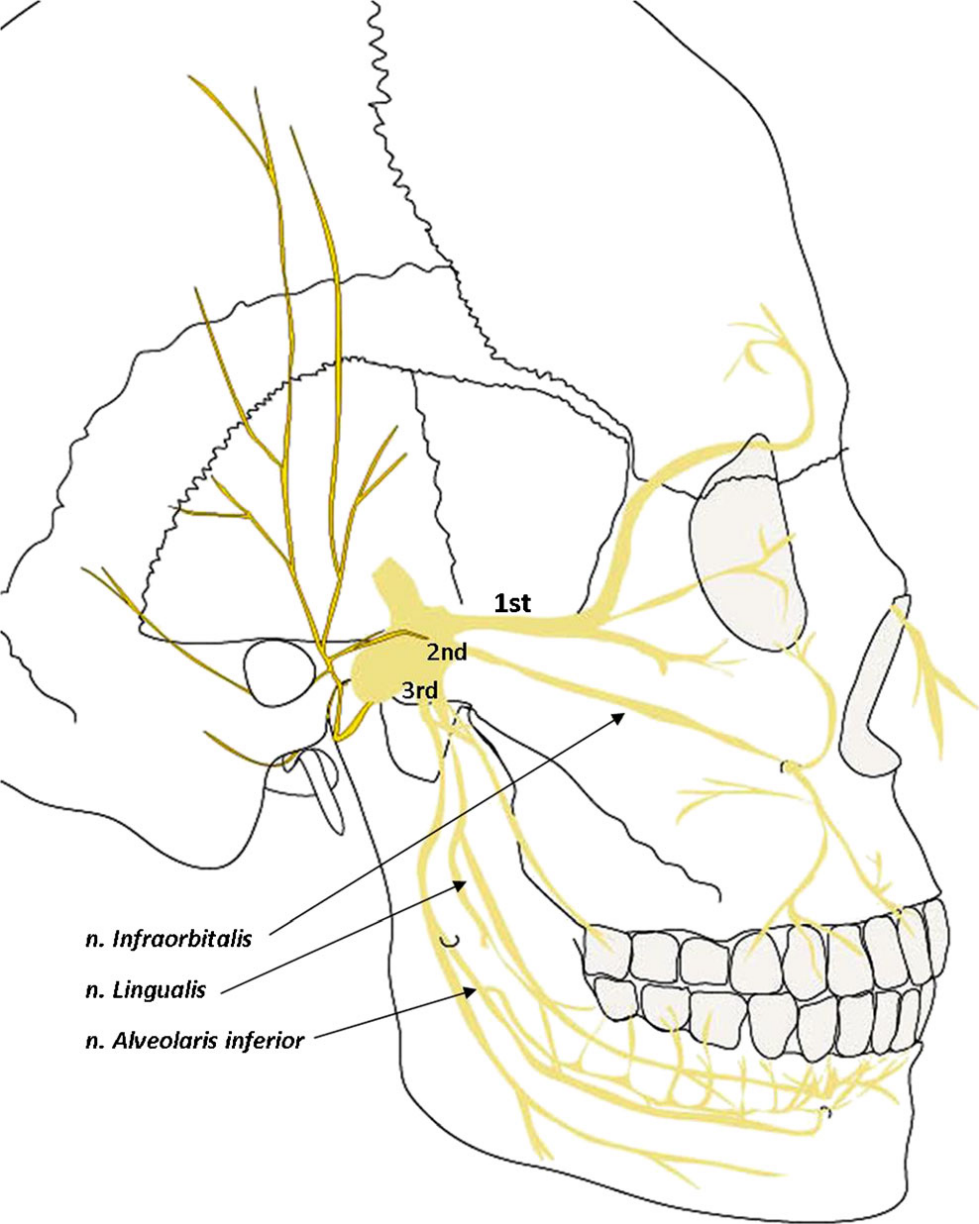
Trigeminal nerve’s sensory distribution in the orofacial area (Illustration Stina Branting)

Traumatic nerve injury of the trigeminal nerve is a major clinical challenge. The frequently most affected trigeminal branch is the inferior alveolar nerve (IAN) followed by the lingual nerve (LN) and finally the infraorbital nerve (ION) [7, 8] (Fig. 1). It has earlier been reported that spontaneous recovery of injured IAN after 6–9 months will leave some degree of long-term permanent disability [9]. Alteration of sensation in the damaged nerve results from either direct or indirect damage due to compression, stretching, or laceration. The degree of alteration depends on the severity of injury [10].

When the nerve is damage, an inflammation process will start which releases a cascade of prostaglandins to the surrounding tissue which will spread to sensory neurons. The inflammation response will maintain the painful symptoms which in turn can develop to both peripheral and central sensitization [11]. During the inflammation process, interactions of macrophages and monocytes from peripheral blood migrate towards the damage site in the peripheral nerve which is connected via cell bodies in the dorsal spinal cord and the trigeminal ganglion and further to central parts of the brain. They will consequently activate microglia, which are surrounded by satellite glia cells (SGCs), and underlie peripheral sensitization which in turn maintain allodynia and hyperalgesia [12]. This finding has been demonstrated in an animal study where injections of capsaicin to the temporal joint capsule showed that SGCs were activated [13]. Further, activation of sensory neurons in the mandible has shown resultant spreading of neuronal activity not only in the third trigeminal branches but also to the first and second branches (Fig. 1). A cross-excitation to extraterritorial sites outside the injured dermatome was the interpretation of the results [14]. Recent evidence in animal studies indicates that deficits of trigeminal nerve system may lead to impairments in learning and memory and neuronal loss in the hippocampus [15].

Orofacial pain induced by trigeminal nerve injury is often a symptom complex rather than a single condition, and it is thought to be caused by multiple factors. However, these factors are poorly understood. A major obstacle in exploring mechanisms and treatments of neuropathic pain is that our conventional understanding of pain physiology and pharmacology has been built primarily on studies of nociceptive pain whereas persistent or neuropathic pain in many aspects differs from, and even is contrary to, nociceptive pain [16]. Clinical research on this problem is difficult as these are not common disorders, and thus, homogenous patient samples for important variables are difficult to obtain. Furthermore, invasive methods often have to be used to address the underlying mechanisms and novel unproven treatments tested.

### Available Treatments

Treatment of abnormal sensation of sensory nerves such as pain is usually, as a first choice, pharmacological. Acute pain can be treated successfully with paracetamol, non-steroidal anti-inflammatory drugs (NSAID), and/or morphine. When the postoperative pain develops into persistent neuropathic pain, medication with antiepileptic drugs such as gabapentin or carbamazepine is used with different outcomes [12]. The side effects of this type of medication can be a problem for the patient, which include drowsiness, dry mouth, and negative mood changes that affect their quality of life.

Searching for new targets in the pharmacological field to treat neuropathic pain is important. Transient receptor potential (TRP) channels are present in sensory neurons and are involved in the development of pain. Neuropathic injury in humans has been shown to increase the expression of TRPA1 [17], and studies on tooth injury have specific shown to increase the expression of the TRPA1 channels [18]. TRP channel antagonists could be promising as novel analgesic agents [19]. There are several antagonists that have been shown to block the TRPA1-induced neuropeptide release in dental pulp. Recently, we have shown that a novel TRPA1 antagonist inhibits TRPA1 agonist-stimulated release of neuropeptides from dental pulp biopsies in an in vivo model [20].

Serotonin (5-HT) is a neuromodulator and plays an important role as a mediator of pain both centrally and peripherally [21]. It has been suggested that 5-HT_3_ receptors are activated in humans with muscle pain [22]. Recently, it was shown from our group that 5-HT_3_ receptors were highly expressed in masseter muscles of women with TMD compare to controls [23]. It was concluded that in myofacial TMD up-regulated 5-HT_3_ receptors could serve as a biomarker. In the search for 5-HT_3_ receptor antagonists as a target for blocking orofacial pain, only one study was found in animals. Painful Injection of formalin to the masseter muscle in rats attenuated nociceptive behavior by both local and systemic administration of a 5-HT_3_ receptor antagonist [24].

Non-invasive treatment modalities such as local anesthesia have been used as neural therapy which approach for long-term relief of pain after nerve injury. Neural therapy should be repeated several times with increasing time intervals. The effect of Local anesthetic has been speculated to provide protection against sprouting in sympathetic nerves [25] and give a pro-inflammatory effect [26]. Recently, a review by Weinschenk raised the question whether or not local anesthetics can interrupt the liberation of pro-inflammatory substances at the terminal plate in neurogenic inflammation. [27].

Another non-invasive type of treatment is low-level laser therapy (LLLT), which has shown good results in subjects with nerve injuries that are identified immediately [28]. For longstanding injuries, there has been registered some improvement with LLLT [29, 30]. In a study, LLLT showed some efficacy for long term of sensory disturbances following third molar surgery [31]. For TMD or temporomandibular joint derangement (TMJD), two systematic reviews have concluded that LLLT is probably more effective for the treatment of TMJD and less effective for TMD [32, 33].

Treatments available for nerve injury have shown some functional recovery in humans, i.e., more sensation and/or less pain, but evidence lacks for nerve regeneration. Surgical procedures for reconstruction of peripheral nerves are available such as microsurgical approaches with direct end-to-end connection. The gold standard for nerve grafting is autologous substrates, sural nerve, or auricular nerve to be used for the trigeminal branches [34]. Disadvantage of this method is donor site morbidity, limited length of available grafts, and potential formation of neuroma [35–37]. Complete recovery is uncommon in all kinds of available treatments today [30]. The limitations of auto-grafting have led to exploration of alternative forms for nerve reconstruction.

### New and Interest Findings

Recently, cell-based therapies have been studied for their potential to enhance peripheral nerve repair. In vivo studies have shown that bone marrow-derived mesenchymal stem cells (BM-MSCs) and adipose-derived stem cells (ADSCs) can physically engraft and myelinate regenerating axons and are comparable to each other [38, 39]. In humans, it is, however, easier to isolate large amount of ADSCs with liposuction than overcoming the discomfort and tissue morbidity associated with bone marrow harvesting [40].

Animal and clinical studies have shown that ADSCs are capable of repairing damaged skeletal tissue [41]. These properties in combination with the large quantity of cells that can be obtained from fat suggest that cells from adipose tissue will be a useful tool in biotechnology and regenerative medicine. ADSCs are commonly characterized by the same methods used for characterizing BM-MSCs: their immunophenotype in the undifferentiated state and their differentiation potential towards the adipogenic, osteogenic, and chondrogenic lineages using specific induction factors. BM-MSCs and ADSCs show very similar expression patterns for surface markers with minor differences. Due to limited publications and the variations in the protocols used, it is very difficult to define the optimal harvesting and isolation techniques. Therefore, the stem cells’ quality must be thoroughly examined prior to the use in clinical applications.

Using biodegradable synthetic material to nerve guidance channels (NGCs) shows promising results [42]. It is important with permeability, swelling, and degradation behavior for the NGCs. Growth-permissive substrate for NGC may include intrinsic scaffolds, which have to be filaments that mimic the fascicular pattern of a nerve.

Neurostimulatory extracellular matrix proteins such as collagen can enhance regeneration. Neurotrophic factors promote neuronal regrowth, sprouting, and new connections between the injured ends of the axons. Growth factors can be fibroblast growth factors (FGF), nerve growth factors (NGF), or neurotrophins (NT 3, 4, 5). Carriers for the growth factors are nanoparticles, microparticles, or hydrogels. The addition of supportive cells in the NGC enhances the regeneration of the nerve axon. Examples of supportive cells are MSCs or neural stem cells. A large number of animal studies have been carried out, mostly in rats and mice. The critical size of the gap between the axons is 10 mm. The choice of analyzing methods for measurement of nerve regeneration employs anatomic and histological methods but a functional evaluation is also necessary. There is still need for optimizing the NGC, especially for the trigeminal nerve, which has so far not yet been well explored. Nanomaterials mimic the properties for natural tissues and may resolve the numerous problems associated with today’s limitations.

Use of mesenchymal stem cells as a new treatment is of interest due to the core properties of these cells. It has been reported that MSCs have an anti-inflammatory effect and they seem to play an important role in nerve healing and regeneration [43]. In animal studies, promising results have been presented where neuropathic trigeminal pain has been reduced following MSC treatment [44••]. Recently, a preliminary reported the outcomes following injection of autologous stem cells into the pain fields in female patients with different diagnosis of neuropathic pain including trigeminal neuralgia, PDAP, and BMS. It was found that the pain intensity scores and use of anti-neuropathic medication were strongly reduced for 6 months after administration of the cells [45••]. It has also been shown that multiple or high doses prolong the therapeutic effect much longer than for a single dose [46•].

### Significant Trends or Developments

To our knowledge, published data on MSC treatment for patient with neuropathic pain are very sparse. In one case study, Ichim et al. (2010) reported a positive result for suppressing neuropathic pain from expanded umbilical cord by intrathecal injection of MSCs [47]. More recently, in a study involving ten patients, Vickers and colleagues (2014) demonstrated a significant effect of MSC treatment on neuropathic trigeminal pain [45••]. They reported that approximately 56 % of patients (5 of 9), who suffered from chronic pain for 4 months to more than 6 years, showed a reduction of pain intensity scores. Moreover, during the investigation, all patients were given anti-neuropathic medication, amitriptyline, and gabapentin. The change in daily dosage requirements of medication showed a near significant reduction in gabapentin and minor reduction in amitriptyline, which indicated a possible biological priority of stem cells in recovery myelinated fibers over unmyelinated fibers. Interestingly, the same group investigators also found that one of the most responders was an eighty-year-old patient. They concluded that MSCs can produce many factors to achieve the therapeutic effect and the secretion profile of the stem cells remains unaffected by age, which is consistent with other observations [48–50]. To a significant extent, the study clarified a positive outcome from neuropathic pain patients in response to a single dose of MSCs, suggesting that a possible enhanced therapeutic effect could be achieved with multiple dosage strategies.

## Conclusion

Patients suffering from nerve injury-induced sensory disturbances and/or pain have greatly reduced quality of life, and it is a big cost for the society. The vast majority of the work on sensory disturbances/pain mechanisms has been carried out in spinal nerve systems. These studies have provided great insight into mechanisms regarding pain of the spinal area. However, it is clear that the pathophysiology of the trigeminal nerve is in many ways different to that found in spinal nerves. Treatments that are available today are not enough to cure the patient, recover the nerve sensibility, and/or reduce pain. Stem cells therapy could be a future solution to solve the situation for the patients.
